# Targeted and controlled anticancer drug delivery and release with magnetoelectric nanoparticles

**DOI:** 10.1038/srep20867

**Published:** 2016-02-15

**Authors:** Alexandra Rodzinski, Rakesh Guduru, Ping Liang, Ali Hadjikhani, Tiffanie Stewart, Emmanuel Stimphil, Carolyn Runowicz, Richard Cote, Norman Altman, Ram Datar, Sakhrat Khizroev

**Affiliations:** 1 Department of Cellular Biology and Pharmacology, Herbert Wertheim College of Medicine, Florida International University, Miami, USAFlorida 33199, USA; 2 Electrical and Computer Engineering, University of California, Riverside, CA 92521, USA; 3 Cellular Nanomed Inc., Weston, FL 33331, USA; 4 Department of Electrical and Computer Engineering, Florida International University, Miami, FL 33174, USA; 5 Department of Obstetrics and Gynecology, Herbert Wertheim College of Medicine, Florida International University, Miami, Florida 33199, USA; 6 Department of Pathology, Miller School of Medicine, University of Miami, Miami, FL 33136, USA; 7 John T. Macdonald Foundation Biomedical Nanotechnology Institute, University of Miami, Miami, FL 33136, USA.

## Abstract

It is a challenge to eradicate tumor cells while sparing normal cells. We used magnetoelectric nanoparticles (MENs) to control drug delivery and release. The physics is due to electric-field interactions (i) between MENs and a drug and (ii) between drug-loaded MENs and cells. MENs distinguish cancer cells from normal cells through the membrane’s electric properties; cancer cells have a significantly smaller threshold field to induce electroporation. *In vitro* and *in vivo* studies (nude mice with SKOV-3 xenografts) showed that (i) drug (paclitaxel (PTX)) could be attached to MENs (30-nm CoFe_2_O_4_@BaTiO_3_ nanostructures) through surface functionalization to avoid its premature release, (ii) drug-loaded MENs could be delivered into cancer cells via application of a d.c. field (~100 Oe), and (iii) the drug could be released off MENs on demand via application of an a.c. field (~50 Oe, 100 Hz). The cell lysate content was measured with scanning probe microscopy and spectrophotometry. MENs and control ferromagnetic and polymer nanoparticles conjugated with HER2-neu antibodies, all loaded with PTX were weekly administrated intravenously. Only the mice treated with PTX-loaded MENs (15/200 μg) in a field for three months were completely cured, as confirmed through infrared imaging and post-euthanasia histology studies via energy-dispersive spectroscopy and immunohistochemistry.

An important challenge in treating cancer in general is to find a technology for a controlled targeted drug delivery and release to eradicate tumor cells while sparing normal cells. The circulatory system can deliver a drug to almost every cell in the body; however, delivering the drug specifically into the tumor cell past its membrane and then releasing the drug into the tumor cells on demand without affecting the normal cells remains a formidable task^1^,^2^,^3^.

Modern research attempts to address this fundamental challenge by using nanoparticles as delivery vehicles^4^,^5^,^6^. Nanoparticles display novel properties due to their (i) unique size, ranging from tens to over one hundred nanometers, to tailor drug delivery into different organs, (ii) wide shape variation, including spheres, rods, and platelets, to help steer the drug-loaded nanoparticles towards more specific targets, and (iii) amenability to comprehensive surface functionalization to meet a wide range of requirements required for conjugation with specific biomolecules and overcoming numerous biological barriers, with or without exploiting the immune system. Last but not least, nanoparticle drug delivery (NDD) shows promise for overcoming the fundamental problem of multidrug resistance (MDR) in cancer therapies.

Such NDD systems rely on using multiple metal and polymer nanostructures, thermally-responsive polymers, electromagnetically (in UV, Visible-Wavelength, and IR ranges) or acoustically activated materials, liposomes, electrochemical processes, and magnetic fields^6^,^7^,^8^,^9^,^10^,^11^,^12^,^13^,^14^. The unique advantages of an external magnetic field control place magnetic nanoparticle systems in a class of their own, especially for the purpose of targeted delivery because they can be remotely navigated to the intended site via application of an external magnetic field gradient^15^,^16^. Systemically administrated nanoparticles have been shown to passively accumulate in a number of tumors because of the enhanced permeability and retention (EPR) effect due to the high leakiness of tumor blood vessels and the lack of a lymphatic system^17^,^18^,^19^,^20^. A small size (<~200 nm but >~10 nm), neutral charge and hydrophilic coating are common prerequisites for successful vascular delivery of cancer drugs. Extremely small particles (<~10 nm) can be removed by the kidney and larger particles (>~200 nm) can be removed by the mononuclear phagocyte system (MPS). Recently, special attention has been given to immunotherapy-mediated active nanoscale approaches. In this case, for example, monoclonal antibodies (mAbs) are used to recognize over-expressed tumor-specific biomarkers, while nanoparticles are used as high-throughput drug carriers^21^,^22^,^23^,^24^,^25^,^26^. Despite the great potential of the nanoparticle delivery, a significant problem remains to ensure that the drug is not prematurely released in the plasma or interstitial space but is released at an appropriate rate once at the intended site, e.g. into the cancer cell cytoplasm^27^. To address this problem, nanoparticles have been formulated to allow for triggering drug release by externally applied temperature^28^,^29^, ultrasound^30^,^31^, intracellular pH^32^, intracellular enzymes^33^,^34^, or the tumor microenvironment^35^. Nevertheless, all these approaches still suffer from inconsistent drug release when the nanocarrier reaches the target. In fact, using NDD systems to control retention and specific delivery of the drug remains a major open question in cancer treatment.

This combined *in vitro* and *in vivo* study shows how a class of multiferroic nanostructures known as magnetoelectric nanoparticles (MENs) can be used to enable externally controlled high-specificity targeted delivery and release of therapeutic loads on demand. Furthermore, such control allows to physically separate the two important functions of drug delivery and release via application of d.c. and a.c. magnetic fields, respectively. The control is achieved because, unlike traditional purely magnetic nanoparticles such as iron oxide nanostructures, MENs display a non-zero magnetoelectric (ME) effect due to strongly coupled magnetostrictive and piezoelectric properties. On the one hand, it is known that cellular membranes are electrically charged and therefore MENs can interact with the cellular microenvironment through electric fields. On the other hand, due to the ME effect, MENs provide a unique way to use magnetic fields to externally control intrinsic electric fields which underlie the chemical bonds between the nanoparticles and the loaded drug as well as the interaction between the drug-loaded nanoparticles and the cellular microenvironment^36^,^37^,^38^,^39^,^40^. It is known that cancer cells differ from their normal counterparts with regards to their electric properties, e.g. membrane potential, cellular dielectric constant, or electric capacitance^41^,^42^. For example, the membrane potential for ovarian cancer cells in interphase is on the order of −3 mV, while it is on the order of −50 mV for the normal counterparts^43^. Hence, the electric-field interaction between MENs and cells differs for cancer and normal cells. Herein, we show how externally-controlled MENs can be used to simultaneously carry a payload of drugs, deliver them to the intended target site, avoiding removal from the circulatory system before they reach the target, enter the cancer cells without affecting the normal cells, and release the drug only after the drug-loaded nanoparticles enter the cancer cells through application of a specific sequence of external d.c. and a.c. fields on demand.

Ideally, the proposed mechanism of drug delivery by MENs can be applied to any cancer; however, for the sake of simplicity, this study has focused on ovarian cancer (OC), which is characteristically metastatic (Stage III) at the time of diagnosis^44^,^45^. Cytoreductive surgery followed by chemotherapy with mitotic inhibitor paclitaxel (PTX) and carboplatin has become the gold standard in OC treatment. Intraperitoneal (IP) delivery through a surgically implanted catheter has shown improved survival rates for OC. Unfortunately, catheter complications and toxicity have precluded widespread adoption of this invasive means of delivery. Therefore, the nanotechnology approach would be a significant advance in OC treatment. Furthermore, this nanotechnology, especially with the use of PTX, could be straightforwardly extended to breast cancer, lung cancer, and pancreatic cancers, among others.

## Results

### Outline of Experiments

This comprehensive study included (i) *in vitro* analyses to investigate the underlying physics of the MEN-based mechanism to deliver and release a drug specifically into cancer cells via application of external d.c. and a.c. magnetic fields, respectively, and (ii) *in vivo* measurements on mice with inhibited immune system (nude mice) bearing SKOV-3 human ovarian carcinoma xenografts to test the hypothesis on animals. The *in vitro* studies included (i) atomic/magnetic force microscopy (AFM/MFM) and scanning tunneling spectroscopy (STS) imaging of the nanoparticles to understand their magnetic-field-dependent electric properties and (ii) detailed AFM/MFM and spectroscopic analyses of the cell lysate content, including MENs and the bioactive drug, under all the investigated conditions of externally applied d.c./a.c. magnetic fields. The experiment allowed to physically separate the d.c-field triggered high-specificity penetration of the drug-loaded MENs into cancer cells and the a.c.-field triggered release of the drug off the MEN carriers into the cells. The *in vivo* studies were conducted to compare the MEN approach with two current state-of-the-art nanotechnology delivery approaches including (i) active approach using polymer nanoparticles conjugated with monoclonal antibodies (mAbs) and (ii) passive EPR-based approach using polymer nanoparticles without any immunoactive reagents. In addition, two groups of mice were studied, including mice in which PTX –loaded MENs were weekly administrated (i) through systemic IV injection into a lateral tail vein and (ii) through localized subcutaneous injection directly into the tumor site grown on the animal’s back, respectively. The tumor progression was monitored through infrared (IR) imaging with IV-administrated mAb-conjugated fluorescent agent Her2Sense 645. After a specimen was sacrificed, the cell morphology in different organs was further studied with hematoxylin and eosin (H&E) stain. Additionally, the same organ tissues were imaged for the tumor presence with the antibody-conjugated fluorescent agent Her2Sense. The biodistribution of the nanoparticles in the tumor sites and different organs of mice treated under different field conditions was studied through the energy-dispersive spectroscopy (EDS) mode of high-resolution scanning electron microscopy (SEM). Finally, after the completion of the treatment, the cured mice were monitored for a period of three months before being sacrificed for further immunohistochemistry and nanoparticle biodistribution studies.

### Physical Properties of MENs

**Figure 1:**
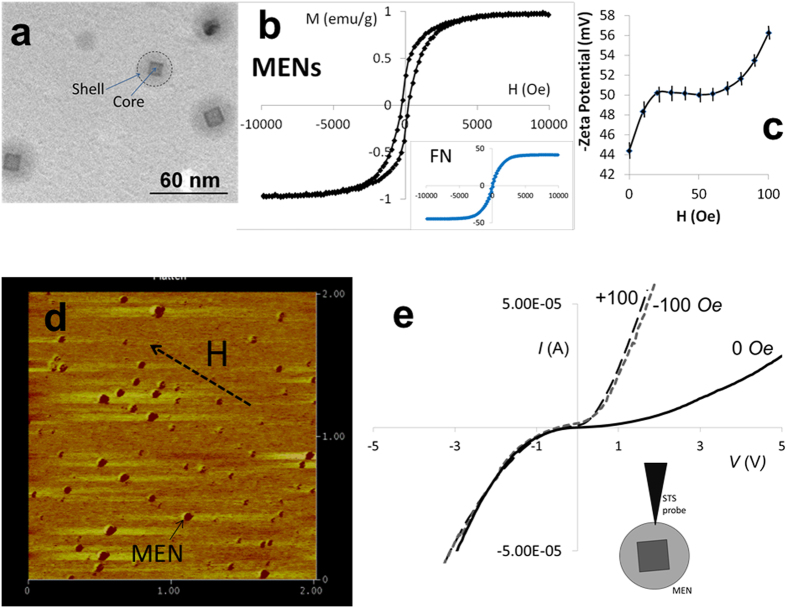
MENs’ physical properties. (**a**) Transmission Electron Microscopy (TEM) image showing the coreshell nanostructure of MENs (spinel CoFe_2_O_4_ core and perovskite BaTiO_3_ shell). (**b**) M-H hysteresis loop of MENs. The insert shows a loop for FNs. (**c**) Magnetic field dependence of Zeta Potential for 0.5 mg of MENs in a 1-ml PBS buffer solution with a pH of 7.3. (**d**) Magnetic force microscopy (MFM) image showing the dipole nature of 30-nm MENs. (**e**) Scanning Tunneling Spectroscopy (STS) I-V curve measured from a point contact between the tungsten nanoprobe of a STM setup and a MEN at three different field values, −100, 0, and 100 Oe, respectively, with a magnetic field applied along the central axis.

MENs with a 30-nm diameter, synthesized through a hydrothermal process, had the coreshell composition CoFe_2_O_4_@BaTiO_3_, which consisted of the magnetic-moment enhancing ferromagnetic spinel core CoFe_2_O_4_ and the magnetoelectricity inducing perovskite shell BaTiO_3_, as shown in a transmission electron microscopy (TEM) image in Fig. 1a 46,47,48,49,50. The mitotic inhibitor PTX was conjugated with the nanoparticles according to the previously described surface functionalization chemistry^40^. The functionalization chemistry is essential to ensure the drug remains strongly attached to the nanoparticles until a special control command is given externally via application of an a.c. magnetic field to release the drug off MENs. It should be noted that the drug is not significantly bioactive while it is attached to the nanoparticles, so it is safe to move the drug-loaded MENs through the circulatory system towards the target site without triggering any toxicity effects^51^. In accordance with this *in vitro* study, a 2-nm thin layer of glycerol-monooleate (GMO) was coated on the MEN’s surface as an intermediate layer between MEN and PTX to provide a specific a.c. release field on the order of 50 Oe (100 Hz). Vibrating sample magnetometry (VSM) hysteresis loops for 30-nm MENs and 30-nm CoFe_2_O_4_ ferromagnetic nanoparticles (FNs) are shown in Fig. 1b. FNs were used as control nanoparticles with a relatively strong saturation magnetization (40 times higher than that for MENs (~1 emu/g)) but displayed no ME effect. This difference between MENs and the control FNs helps understand the different roles of electric and magnetic fields in the studied delivery mechanism. The magnetic field dependence of the measured Zeta Potential (ZP) for 0.5 mg of MENs in a 1-ml PBS buffer solution with a pH of 7.3 (similar to that of blood) is shown in Fig. 1c. It can be noted that the d.c. magnetic field variation from 0 to +/−100 Oe resulted in an increase of ZP by over 30%; moreover, ZP value depended only on the field strength and didn’t depend on the field orientation. ZP reflects the established equilibrium surface charge of the nanoparticles due to the double-layer chemistry in a liquid solution. Because this charge would interact with electrically charged cell membranes, the value of ZP affects the degree of the cellular uptake of the nanoparticles. Therefore, the finding of the relatively strong magnetic field dependence of ZP is an important discovery for enabling an externally-controlled targeted delivery mechanism. A typical Magnetic Force Microscopy (MFM) image of naked MENs (deposited on a silicon oxide substrate) is shown in Fig. 1d. Before taking an image, MENs were magnetized along an in-plane direction by an external biasing field H (of 100 Oe); a direction in which each MEN’s MFM image contrast vari s from darker to lighter reflects the dipole orientation along the field direction. A Scanning Tunneling Spectroscopy (STS) I-V curve measured from a point contact between the tungsten nanoprobe of a Scanning Tunneling Microscopy (STM) setup and a MEN at three different field values, −100, 0, and 100 Oe, respectively, with a magnetic field applied along the central axis, is shown in Fig. 1e. The STS curves indicate that the effective conductivity of the nanoparticles increases with application of a d.c. magnetic field. Similar to the above ZP measurements, the effect doesn’t depend on the field orientation.

### Direct Measurements of Cell Lysate Content as a Function of D.C/A.C.-Magnetic Field Application Via Scanning Probe Microscopy and Spectrophotometry

**Figure 2:**
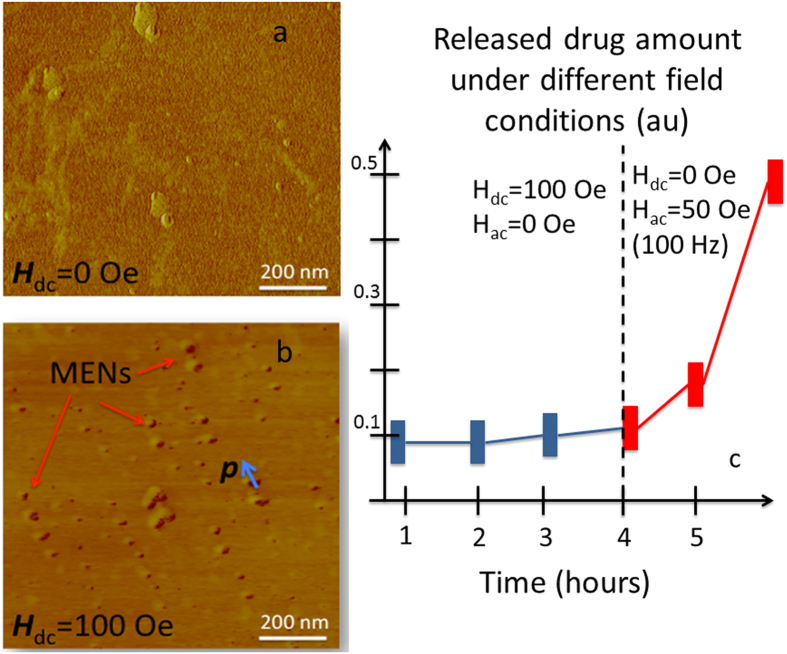
Cancer cell lysate content as a result of ac/dc-field application. MFM images of the cancer cell lysate content (**a**) before and (**b**) after the d.c. field (H_dc_ = 100 Oe) application. The red arrows indicate the location of MENs. The blue arrow shows the orientation of the MEN’s dipole field. (**c**) Spectrophotometrically measured bioactive drug amount inside the cells (normalized per cell protein amount) under different a.c./d.c.-field conditions. The amount of the bioactive drug per protein content is measured at a 230-nm absorption wavelength.

The purpose of this *in vitro* experiment was to directly confirm (i) the penetration of drug-loaded MENs into the target cells as a result of the d.c.-field application and (ii) the release of the drug inside the target cells as a result of the a.c.-field application. Again, it should be noted that the controlled drug release after the drug-loaded MENs penetrate the cancer cells is essential for increasing the drug bioactivity^51^. The drug was conjugated with MENs, and the target cells were prepared according to the procedures described in detail in our previous publications^36^,^40^. The new procedures, which are related to the cell lysate preparation, i.e., identification of MENs inside cells and spectrophotometric measurements of the drug content under different magnetic field conditions, are described in detail in Section *Methods and Procedures*. MFM images of the cancer cell lysate obtained from the cell media with MENs before and after application of a 100-Oe d.c. magnetic field are shown in Fig. 2a,b, respectively. While no MENs were detected in the lysate prior to the d.c.-field application, strings of MENs were clearly observed after the field application. Unlike other lysate “debris”, MENs are characterized by clearly identified dipolar field polarization in the MFM image, as highlighted by the blue arrow. The image shows the polarity reversal within each MEN as a transition from dark to light along the orientation of the dipolar field. “Zoomed out” AFM and MFM images of the same lysate samples are shown in supplementary materials Fig. S1A. The spectrophometrically measured absorption wavelengths and standard linear calibration curve used to define the amount of the drug per unit protein are shown in supplementary materials Fig. S1B. According to the absorption spectrophotometry analysis, the bioactive drug is characterized by a maximum at a 230-nm wavelength. The dependence of the released drug amount measured inside the cells on the application of d.c/a.c. magnetic fields is summarized in Fig. 2c. No significant amount of the drug could be detected in the cancer cells after the application of a 100-Oe d.c. field, although the drug-loaded nanoparticles were detected inside the cells. Only after the application of a 50-Oe 100-Hz a.c.-magnetic field, substantial amount of the drug was detected inside the cancer cells.

### *In Vivo* Studies

#### Weekly Treatment Through Intravenous and Subcutaneous Injections

It took approximately four to six months to grow a tumor size of over 200 mm^3^ through inoculation of approximately 1mln human ovarian carcinoma SKOV-3 cells on the right flanks of nude mice under treatment. Only after the tumor had reached the critical volume of over 200 mm^3^, the mouse was treated with weekly injections of PTX loaded on either 30-nm MENs or one of the three control nanocarriers: (i) poly(lactice-co-glycolic acid) (PLGA) nanoparticles (Supplementary Materials Fig. S2)^52^ conjugated with an active reagent in the form of a monoclonal antibody (mAb) (PLGA-mAb), (ii) pure PLGA nanoparticles without any active targeting reagents, or (iii) 30-nm FNs, with a relatively high saturation magnetization but without any ME effect. The humanized mAb that targets the human epidermal growth factor receptor-2 (HER2), which is overexpressed in the ovarian tumor cells, was used as the antibody in the PLGA-mAb nanocarrier. Each treatment sub-group was tested on two mice.

The first *in vivo* experiment was motivated by the above basic study results and the previous *in vitro* studies which had demonstrated that the application of an external a.c. magnetic field after application of a d.c. field had a substantially more pronounced effect on the drug release compared to that of an external d.c. magnetic field alone^36^,^39^,^40^. In the *in vitro* experiment, it took only a few minutes to release most of the drug carried by MENs on demand via application of a relatively weak (~50 Oe) and low-frequency (~100 Hz) a.c. field. For comparison, it took several hours to release the same amount of drug by the same amount of MENs via application of a 100-Oe d.c. magnetic field. In the current *in vivo* experiment, the above PTX-nanocarrier formulations were intravenously injected into a tail vein with a weekly dose of approximately 15 μg of PTX and an adequate amount of either of the three nanocarriers, MENs (~200 μg), PLGA-mAb (~200 μg), or PLGA (~200 μg), respectively, in a 20-μl saline solution. By default, when MENs were used as the nanocarriers, right before each injection, a high-moment neodymium magnetic coin was attached with a 3M^TM^ Vetbond^TM^ tissue adhesive over the tumor site and kept attached for approximately one hour after the injection, as shown in Supplementary Materials Fig. S3A. This neodinium magnet, with a magnetic anisotropy normal to the plane and a saturation magnetization of 800 emu/cc, could generate a d.c. field on the order of 100 Oe a few millimeters away from the surface. Therefore, the effect of the magnet was equivalent to application of a local 100-Oe external d.c. magnetic field immediately after each weekly administration of PTX-loaded MENs. An a.c. field of 50 Oe at 100 Hz was generated approximately 24 hours after each injection by placing a mouse under study in a special cage, with a set of 4 electromagnetic coils under its floor, as shown in Supplementary Materials Fig. S4. Control sub-groups included mice treated by the same amount of PTX-loaded MENs without application of a magnetic field.

**Figure 3:**
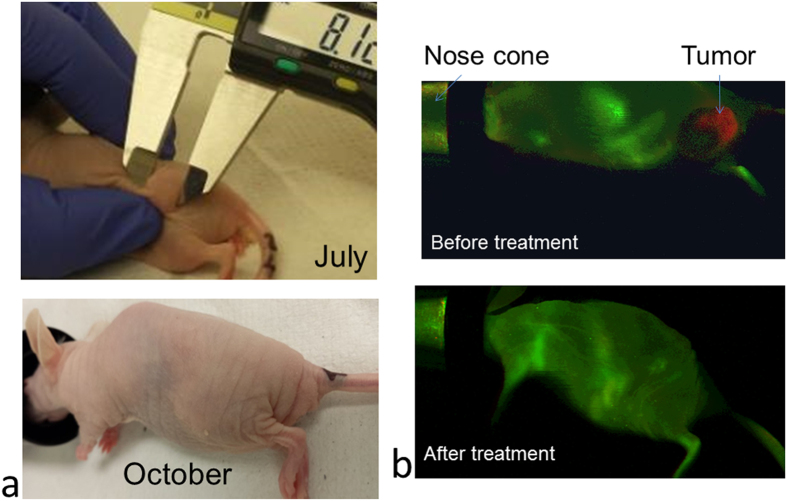
A cured mouse cured throughout the treatment. (**a**) Tumor photographs at its peak on July 11 (268 mm^3^) and on October 13 (no visible tumor). (**b**) IR images (with fluorescent agent Her2Sense 645 taken before (top) and after the completion of the MEN treatment. The agent had excitation and emission maxima at 643 and 661 nm, respectively.

According to the current results, only the mice which were subjected to the magnetic field treatment following each weekly injection of PTX-loaded MENs were completely cured of the tumor after approximately 3 months of weekly IV injections. Photographs of the tumor protrusion in one of the treated mice during its peak size in the beginning of the treatment (July) and at the end of the treatment (October) is shown in Fig. 3a. The progression is presented in more detail in Supplementary Materials Figs S3B–E. The tumor size had been recorded daily since July 11, when the tumor size was approximately 268 mm^3^ while the mouse weighed about 0.8 g and showed relatively passive behavior characteristic of a sick mouse. On August 14, the mouse weighted 0.9 g and the tumor size was reduced down to 51 mm^3^. On September 5, the tumor further shrank to 20 mm^3^. On October 13, the weight remained 0.9 g and no tumor could be detected. The last weekly dose was given on December 13. After December 13, the mouse had been in a stable condition until it was euthanized for further immunohistochemistry and particle biodistribution studies on February 17, 2015.

In addition, mice were monitored monthly through IR imaging with Her2Sense 645 fluorescent agent (with excitation and emission maxima at 643 and 661 nm, respectively, administrated through IV injections in the tail. Each dose of the agent in solution was supplied in 100 μL of 0.02 M histidine, 0.02 M NaCl, 5% sucrose pH 6.0, approximately 10 hours before an imaging session to maximize the imaging signal. IR images at the mature stage of fully grown tumor before treatment and after completing the 3-month treatment are shown in Fig. 3b (top) and (bottom), respectively. The same images are shown in color in the Supplementary Materials Fig. S3F.

**Figure 4:**
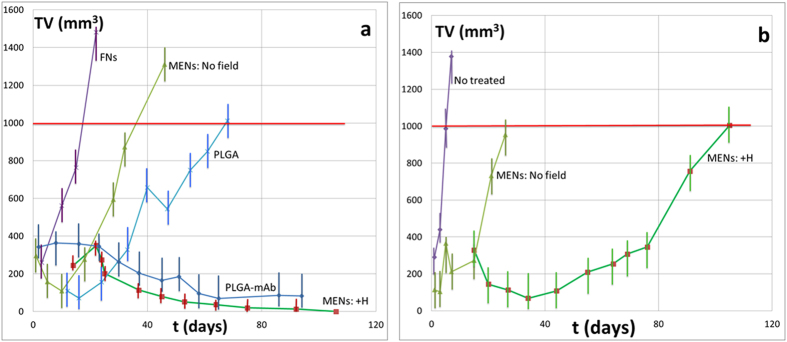
Comparison of systemic IV and localized subcutaneous administration. (**a**) Tumor volume (TV) in response to IV administration of PTX by MENs and control nanocarriers at different field conditions. The dotted line shows the critical tumor size. (**b**) TV in response to subcutaneous administration of PTX by MENs with and without a local magnetic field application.

Time dependencies of the tumor size in mice treated weekly through IV administration of PTX loaded on the four nanocarriers including MENs, FNs, PLGA, and PLGA-mAb, respectively, are plotted in Fig. 4a. The two types of MENs reflect weekly treatments with and without the magnetic field treatment, respectively. On average, two mice were used for each parameter setting.

Alternatively, MENs and other nanocarrier counterparts were comparatively studied in mice treated weekly through subcutaneous administration of PTX loaded on MENs with and without local application of magnetic fields, as shown in Fig. 4b. A control mouse, which was not treated, is also shown. On average, two mice were used for each parameter setting.

#### Nanoparticle Biodistribution: Detection of MENs in Different Organs and Tumor Sites via Energy-dispersive Spectroscopy (EDS)

**Figure 5:**
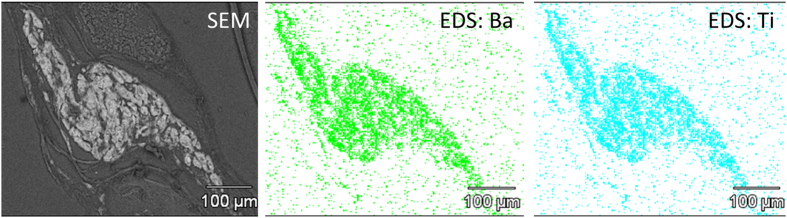
SEM-EDS detection of MENs in the tumor site of a mouse at an initial treatment stage. (left) Regular SEM, (middle) Barium SEM-EDS and (right) Titanium SEM-EDS.

To understand how an external magnetic field could enable a controlled high-specificity targeted delivery of MENs, the following nanoparticle biodistribution study was conducted. To measure the biodistribution of MENs in different organs as well as to confirm the presence of MENs in the tumor sites under different field conditions, a detailed spectroscopic analysis of tumor and organ sections post euthanasia was conducted using energy dispersive spectroscopy (EDS) with high-resolution scanning electron microscopy (SEM). An SEM image of a tissue section with a tumor region from a mouse at an initial treatment stage (two weeks after the first weekly injection), when the tumor size still remained relatively high (~250 mm^3^), is shown in Fig. 5 (left). The treatment was conducted with the basic MEN-PTX nanocarrier-drug combination according to the default magnetic-field treatment. SEM-EDS images of barium and titanium regions in the same tissue section are shown in Fig. 5 (middle) and c (right), respectively. Barium and titanium are the key compositional elements of MENs. These images prove that MENs were indeed localized in the tumor sites. A detailed spectroscopic chemical composition in the tumor is shown in Supplementary Materials Fig. S5A.

However, when a mouse was treated with the same dosage of PTX-loaded MENs, but without application of an external magnetic field, significantly fewer MENs (approximately by a factor of five) were detected in the tumor site although the nanoparticles were normally present in different organs including liver, lung, spleen, and kidney. The two charts in Supplementary Materials Fig. S5 illustrate the relative concentrations of MENs in these organs as well as in the tumor site in three euthanized mice under study including a cured mouse after the three-month recovery period and two positive control mice, which were treated only for two weeks with and without the magnetic field application, respectively. Such a relatively short treatment for the two control mice was chosen to understand the nanoparticle biodistribution while the tumor size remained at its peak value. H&E-stain cell morphology imaging and Her2Sense-antibody-specific imaging of tissue sections of cured and control mice confirmed the presence of metastasis (in kidney) in positive control mice with untreated tumor and complete eradication of both primary and metastasized tumor in the cured mice (Supplementary Materials Figs S6 and S7).

## Discussion

Like conventional magnetic nanoparticles, e.g., FNs and their superparamagnetic phase variants (with superparamagnetic iron oxide nanoparticles (SPIONs) being the most popular form), MENs have a non-zero magnetic moment and therefore can be transported in the circulatory system via application of an external magnetic field with a non-zero spatial gradient and also imaged via existing magnetic imaging techniques including magnetic resonance imaging (MRI) and the recently emerged magnetic particle imaging (MPI) technique, with the sensitivity at least an order of magnitude better than that of state-of-the-art MRI systems^53^,^54^. However, unlike MNs, MENs offer a new and far-reaching functionality, which is an energy-efficient control of intrinsic electric fields in the vicinity of MENs via application of external magnetic fields. This new functionality is a result of the strong ME coupling in this new class of nanostructures due to inherently paired magnetostrictive and piezoelectric effects. As a result, MENs introduced in a biological microenvironment act as localized magnetic-to-electric-field nano-converters that allow an external control of the electric signals that underlie the intrinsic molecular interactions between specific cells and the drug-loaded MENs as well as the interaction between MENs and the loaded drug. An immediate consequence of this capability is the freedom to engineer an adequately strong bond between the drug and the nanoparticles to avoid the undesired release of the drug before it reaches the intended site; only when an a.c. magnetic field is applied, this strong bond is “turned off” on demand. In addition, the MEN’s new capability to control local electric fields remotely opens an exciting and previously unexplored path to exploit the intrinsic electric properties of the cell membrane to enable an electric-field mediated targeted and high-specificity delivery. Indeed, due to the presence of ion channels and other electric-field driven properties, the cell membrane is an electrically polarizable medium. As a result, its properties can be significantly and differently (for normal and tumor cells) affected by an electric field. For example, in general, the tumor cells have lower values of the membrane potential and more importantly, substantially lower values of the electroporation threshold electric field compared to those for normal cells^43^.

**Figure 6:**
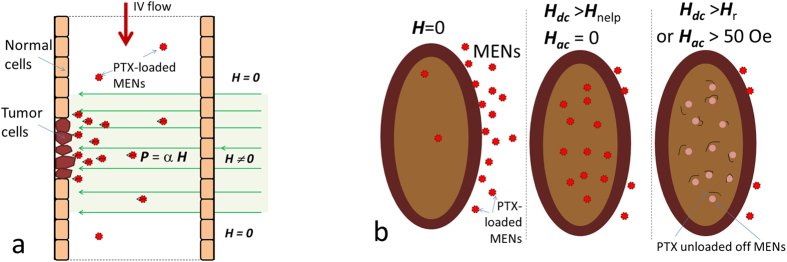
Illustration of a field-controlled targeted drug (PTX) delivery by MENs through a capillary. PTX-loaded MENs are administrated through an IV injection into the circulatory system. (**a**) Applying a local magnetic field, ***H***, enhances the targeted delivery efficacy by localizing MENs into the tumor region. In addition, due to the non-zero ME coefficient, α, the magnetic field polarizes the MEN’s electric dipole, ***p***, and simultaneously increases the negative surface charge as shown in this study. Due to the magnetic-field-induced MENs’ surface charge and the fact that cancer cell membranes undergo a transition during which their conductivity temporarily increases, the induced electric force steers MENs towards the cancer cells. (**b**) When PTX-loaded MENs approach the surface of the tumor cell membrane, they are transferred inside the cells due to the high-specificity nanoelectroporation effect, if the d.c. field, H_dc_, is above the nanoelectroporation threshold field for the cancer cells, H_netf_c_, but less than that for the normal cells, , H_netf_n_, Then, as a 100-Hz a.c. field of ~50 Oe is applied, PTX is released off MENs inside the cancer cells. The normal cells remain intact because their nano-electroporation threshold fields are substantially higher.

Unlike active delivery approaches, e.g. using mAbs, MENs’ delivery does not require an immuno-sensitive targeting agent. On the contrary, MENs offer a passive delivery mechanism, which has a complementary component to the well-known EPR effect. The physical targeting mechanism is driven by the MENs’ intrinsic electric fields, which in turn can be externally controlled via application of magnetic fields. Again, these intrinsic electric fields determine (i) the strength of the MEN-drug bond and (ii) the interaction between the drug-loaded MENs and the cell membranes. Here, it makes sense to define a specificity factor, SF, so that SF = *P*_cancer_/*P*_normal_, where *P*_cancer_ and *P*_normal_ stand for the probabilities to deliver the drug into the cancer and normal cells, respectively. It can be reminded that in case of the EPR effect, the specificity factor is higher than 1 due to the higher leakiness and stronger retention of the cancer cells compared to the normal cells. Indeed, fenestrations in the cancer cell membranes (to punctuate the microvasculature) are larger than approximately 200 nm in diameter, compared to approximately 50-nm fenestrations in the normal cells; as a result, the specificity can be achieved by using nanoparticles with a size range between 50 and 200 nm^55^. In addition, after the nanoparticles enter the tumor sites, they tend to be retained there for longer time because of the degraded lymphatic system. Although the EPR effect helps deliver the drug-loaded nanoparticles into the tumor aggregates, it doesn’t ensure their delivery into the cancer cells themselves. As for the MEN’s delivery, because of the existence of an externally-controlled surface charge, MENs bring another component to targeted delivery not only to further increase the specificity factor by ensuring the nanoparticles carry the drug across the cancer cell membranes only, without affecting the normal cells, but also to provide an entirely new function of controlled drug delivery an drelease. Both the MENs’ electric charge and dipole generate electric fields; the electric field from the charge has a more significant effect because it drops with a distance much slower (~1/r^2^) compared to the field from the dipole (~1/r^3^). (Again, it is worth reminding that the electric charge, reflected in a non-zero negative Zeta potential, exists due to the presence of double layer around the surface of a MEN in the circulation solution, i.e. blood or lymph, according to the colloidal chemistry. As for the dipole field, it exists because of the ME effect. Naturally, both charge and dipole electric fields grow with application of an external magnetic field). In turn, because the cell membranes are also electrically polarized, this net electric field acts on the cell membranes; it is commonly believed that the cell membranes have a negative electric charge if the pH level of the microenvironment is basic (like in the blood)^56^. Furthermore, the electric properties of the cancer and normal cells of the same type are different because of the different membrane morphology^57^,^58^. Therefore, because MENs allow to use an external magnetic field to control this intrinsic electric field by controlling the charge (Fig. 1c,e), the following two-step process for drug delivery and release can be enabled via application of d.c. and a.c. magnetic fields, respectively, as described below in more detail and illustrated in Fig. 6a,b.

Step 1 (Application of a d.c.-magnetic field to trigger the high-specificity delivery, in addition to that due to the EPR effect) Because both MENs and cell membranes are negatively charged they repulse from each other. Because of this repulsion force, MENs can easily go through a capillary without being engulfed by the surrounding cells. The repulsion electric field due to the charge stored on the membrane can be on the order of 100 V/cm, depending on the charge amount. However, when a MEN is sufficiently close to the cell membrane surface, its electric field (on the order of 1000 V/cm, mostly due to the surface charge) is adequately strong to induce local nanoelectroporation of the cancer cells but not too strong (<~5000–10,000 V/cm) where it may cause this effect in the normal cells^38^,^40^,^41^,^42^. The nanoelectroporation occurs as an intermediate local rearrangement in the cellular membrane’s lipid bilayer due to application of a sufficiently large electric field; it is known that such a field-dependent rearrangement leads to cellular uptake of drug-loaded nanoparticles near the surface of the membrane^38^. According to one model, during the intermediate increase of the membrane’s conductivity (a sort of electric breakdown at a threshold field), the conducting surface acts as an electric “mirror” which in turn induces a local attraction force between MENs and the cancer cells, F_mirror_ = *k*Q^2^/4*r*^2^, where *Q* is the MENs’ electric charge and *r* is the distance between the nanoparticle and the membrane surface, and the Coulomb’s Constant *k* = 8.988 × 10^9^ (Nm/C^2^). Q can be derived from the above ZP measurements. Considering the average MEN’s diameter *d* = 30 nm, and ZP *V* = −44 mV, the MEN’s average effective surface charge would be *Q* = *Vd*/*k* = ~−1.5 × 10^−19^ C. It can be noted that this value, obtained through the back-of-the-envelope analysis, is comparable to the charge of a single electron. To zeroth-order approximation, one can find the critical distance between the nanoparticle and the membrane surface, r_c_, below which the attraction electric field would be above the electroporation threshold on the order of 1000 V/cm, *r*_*c*_ = 0.5(*kQ*/*E*)^0.5^ ~ 50 nm. As found in this study (Fig. 1c) the surface charge of MENs and consequently the specificity factor can be further increased under application of a magnetic field. For example, application of a 100-Oe d.c. field increases the MEN’s surface negative charge to ~−1.9 × 10^−19^ C and therefore the cutoff distance to approximately 70 nm away from the membrane’s surface. In turn, this would increase the number of the nanoparticles capable of triggering local nanoelectroporation. Because of the laminar flow of the blood in a capillary, the exact dependence would be highly non-linear. Ideally, an adequately high magnetic field should be applied to induce a MEN’s charge sufficiently high to generate an electric field above the electroporation threshold field from any point inside the capillary. According to this analysis, for this particular set of parameters, this field would approximately range from 100 to 1000 Oe. The described process is illustrated in Fig. 6a.

Step 2 (Application of an a.c.-magnetic field to trigger the drug release on demand) As the next step, release of the drug inside the cancer cells can be triggered via application of an a.c. external magnetic field. In this case, even a relatively small magnitude a.c. field (~50 Oe) at a frequency on the order of 100 Hz is sufficient to release significant amount of the drug inside the cells^36^,^39^. It is imperative to release the drug off MENs to increase the bioactivity of the drug^51^. The described process is illustrated in Fig. 6b. Here, it can be mentioned that the near-d.c. frequency range under study, on the order of 100 Oe, is orders of magnitude smaller than the frequency which would be applied to conventional iron-oxide based nanoparticles to trigger any heat-dissipation effects, not to mention that the saturation magnetization for MENs is also significantly smaller (by a factor of 40) than that for the iron oxide nanoparticles. Therefore, thermal effects, which are proportional to the frequency and the saturation magnetization squared, cannot be detected in this case. Further, it should be understood that at such a small amplitude and a relatively low frequency, even a 12-hour long application of the a.c. field is not going to cause any nanoparticle internalization by normal cells because the energy provided by the field is not sufficiently high to overcome the barrier due to the normal cell membrane.

Indeed, these combined *in vitro* and *in vivo* studies on nude mice with xenografted ovarian carcinomas for the first time demonstrated that MENs, a new class of multiferroic nanoparticles in medicine, could enable a mechanism to independently control high-specificity targeted drug delivery and release. By directly measuring the lysate content, including the presence of MENs through AFM/MFM imaging and of the drug through absorption spectrophotometry, it was proven that the application of a d.c. magnetic field (~100 Oe) provided targeted high specificity delivery into the cancer cells, while the subsequent application of a low-frequency a.c. magnetic field (~50 Oe, 100 Hz) ensured high-efficacy release of the drug (Fig. 2). Indeed, the particles were found inside the cells (through direct AFM/MFM scanning) only after the application of a d.c. field, while the amount of the “free” drug was substantially increased (as found through spectrophotometry analysis) only after the application of an a.c. field. Again, it is critical to release the drug off the carrier nanoparticles, i.e. MENs, to increase the bioactivity of the drug. The a.c.-field application does exactly this; it releases the drug off MENs on demand only after it is transferred into the target cancer cells via the preceding d.c.-field application. In other words, MENs allow for a two-step external control to physically separate the two important functions of the high-specificity targeted drug delivery and release, respectively. The provided capability to precisely time the release of the drug, or, in other words, to control the drug’s retention, is important not only for targeted drug delivery in cancer treatment but also in any other medical field where timed drug delivery is key. In addition, the externally controlled delivery/release mechanism, which is independent of the immune system, provides flexibility in surface functionalization to adequately attach the drug to the nanoparticles for avoiding the drug’s loss or degradation while it is being transferred through the circulatory system to the target site. For comparison, with the current approaches the drug cannot be attached to the nanoparticles with an adequate strength to avoid a significant loss before it reaches the target, because the release in these approaches is typically expected to be activated by only a slight difference in the cellular microenvironment at the target site, e.g. due to a different pH level, etc.

The mice under study were treated through weekly injections of PTX loaded on either 30-nm MENs or one of the three control nanocarriers, (i) PLGA nanoparticles conjugated with HER2-neu monoclonal antibody (PLGA-mAb), (ii) pure PLGA nanoparticles without any active targeting reagents, or (iii) pure magnetic nanoparticles (30-nm FNs) with a relatively high saturation magnetization (~40 emu/g) but without any ME effect. Only the mice which were subjected to weekly IV injections of PTX-loaded MENs immediately followed by application of a local magnetic field were completely cured of the cancer after approximately three months of treatment. For example, for one specimen, the tumor size was reduced from its peak value of approximately 270 mm^3^ to being completely absent (Fig. 3a). In parallel, the tumor development was monitored through IR imaging using monthly IV injections of Her2Sense 645 fluorescent agent in the tail. The IR imaging confirmed the complete disappearance of the tumor by the end of the three-month treatment (Fig. 3b). For three months after the last injection, the mouse had maintained an active life style characteristic of a healthy specimen. After the three-month period, it was euthanized for immunohistochemistry and nanoparticle biodistribution studies. The following H&E-stained cell morphology and Her2Sense-antibody-stained tissue imaging studies showed no trace of cancer cells in any of the organs of the field-treated mice. On the contrary, both imaging studies performed on positive control mice clearly showed presence of cancer cells not only directly in the tumor site but also in other organs, e.g. kidney, reflecting a metastatic development of the cancer (Supplementary Materials Figs S6 and S7).

The study indicated a strong dependence of the treatment on the application of a local magnetic field after each injection (Figs 3, 4, 5). This was consistent with the hypothesis that a local magnetic field could improve targeted delivery. Again, only the mice which were treated weekly through IV administration of PTX-loaded MENs with a field application following each injection were cured of the cancer. The fact that the purely magnetic nanoparticles, FNs, despite their substantially higher saturation magnetization, 40 emu/g versus 1 emu/g for MENs, didn’t perform well, indicated the importance of the ME effect in MENs, in agreement with our previous theoretical and *in vitro* studies. Evidently, the magnetic field localized the nanoparticles in the region of the tumor; moreover, the electric field, locally induced by an external magnetic field due to the ME effect, ensured that the nanoparticles penetrated the cancer cells, as confirmed through a SEM/EDS analysis (Fig. 5). The study proved that a d.c. magnetic field on the order of 100 Oe, which could be generated by a neodymium magnetic coin attached to the tumor side of the mouse approximately one hour, was sufficient to induce cell penetration by PTX-loaded MENs and then eventually allow the drug to be released inside the cell via application of a 50-Oe 100-Hz a.c. field. If a substantially higher field had been applied, not only could the drug have been prematurely released but also the chances to deliver and release the drug into the normal cells could have been more significant. In addition, it should be observed that the control mAb-mediated nanotechnology delivery couldn’t match the MEN approach. Although the mAb delivery indeed substantially slowed down the tumor progression, it couldn’t completely eliminate the tumor, while complete elimination of the tumor could be achieved through the field-controlled MEN delivery. Finally, although a field application has an effect, it could be noted that even without any field treatment, the mice treated with PTX-loaded MENs had a better survival rate, compared to that of the non-treated mice or of the mice treated with PTX-loaded FNs (Fig. 4). This could be explained by the MEN’s remnanent magnetization, which in turn should result in a remnanent electric dipole moment.

A comparison between systemic IV and localized subcutaneous injections of PTX-loaded MENs showed that although both delivery approaches could significantly slow down the progression of the tumor, the IV administration was more effective and thus, unlike the subcutaneous administration, could completely eradicate the tumor during the three-month treatment period (Fig. 4a). This result was in agreement with a hypothesis that the physical mechanism for MENs to target cancer cells was the Coulomb force. In other words, the underlying mechanism was due to magnetically-controlled electricity due to the ME effect rather than due to pure magnetism. As explained above, the Coulomb force was attractive due to the electric charge “mirror” effect experienced by the nanoparticles when they passed by the cancer cell membranes (due to the intermediate local increase of the cell membrane’s conductivity during the electroporation process), provided the electric field by the particles was above the electroporation threshold value for the cancer cells (Fig. 6). The targeting force could be further increased when an external magnetic field was applied due to the discovered field dependence of MENs’ Zeta potential in the blood (Fig. 1c). As described above (Fig. 6), when the charge is increased via application of a magnetic field, more MENs ended up in a sufficient proximity to the cancer cell membrane surface to be able to trigger a local electroporation effect which in turn caused a greater amount of the drug to transfer into the cancer cells, compared to the subcutaneous administration, in which MENs had limited mobility.

An important observation from this study was the strong dependence of the number of MENs in the tumor site and various organs of treated and control mice on an external magnetic field. The nanoparticle biodistribution was measured with a relatively high precision based on their chemical composition using the EDS mode of high-resolution SEM performed on tissues from different organs post euthanasia. Under the default treatment conditions, at an early treatment stage, when the tumor size was still at its peak value (of above 200 mm^3^), the nanoparticles were found to be localized in the tumor site (Fig. 5). However, when the same type of weekly treatment was performed without any field application, significantly fewer nanoparticles were found in the tumor site, although they were found in different organs according to the usual distribution pattern (Supplementary Materials Fig. S5). This observation was consistent with our hypothesis that high-specificity targeted delivery could be controlled via application of an external magnetic field. In summary, the application of a d.c. magnetic field results in non-linear effects and could produce a number of functions including (i) localization of the nanoparticles into a region close to the tumor site if the magnetic field is applied locally, (ii) induction of an electric dipole moment through the ME effect (Fig. 1d), and (iii) the resulting increase of the MENs’ negative surface charge (Fig. 1c). The first function (localization) could be produced also by FNs because they also had a non-zero magnetic moment, not to mention, of a substantially higher strength (by a factor of ~40) compared to that of MENs (Fig. 1b). The fact that despite the high moment, FNs, unlike MENs, could not produce a comparable quality delivery reflected the significance of functions (ii) and (iii) for achieving field-controlled delivery. These two functions could be achieved only with MENs. In addition, the EDS study showed that substantially fewer nanoparticles were found in organs of the cured mice (three months post treatment), compared to those of the positive control counterparts, which were treated only for two weeks with and without application of a magnetic field, respectively, while the cured mice were treated for three months and then allowed to recover for additional three months without any treatment (Supplementary Materials Fig. S5). It is likely that the three-month recovery period was sufficient for most nanoparticles to be excreted from the system. Also, in general, only a small fraction of the nanoparticles had accumulated in the kidney. Coincidentally, in positive control mice, which were treated in the field environment only for two weeks, metastasized cells were found (through H&E staining of tissues) in the kidney only (Supplementary Materials Fig. S7).

The fact that significantly fewer nanoparticles were detected in the tumor site when no external magnetic field was applied after each injection indicated that the nature of the MEN-based targeting was passive. However, it is different from the well-known EPR effect. For example, the same size FNs, which carried the same amount of drug, didn’t achieve the same degree of localization as MENs did in the tumor site, although both FNs and MENs experience both localizing forces, due to the d.c. magnetic field and EPR effect, respectively. Not to mention, because of the higher saturation magnetization (~x40), the localizing magnetic field acting on FNs is significantly higher than that acting on MENs, which could have only further promoted the accumulation in the tumor due to the EPR effect. Instead, it is more likely that the nature of the MEN-based high-specificity targeting was affected by the local electric-field interaction between MENs and the cancer cells, which in turn was controlled by an external magnetic field due to the magnetoelectric effect and induced surface charge, according to our hypothesis (Fig. 6). Obviously, these magnetic-field triggered effects are non-linear, as can be noted, for example, from Zeta potential measurements (Fig. 1c).

On a final note, it is worth mentioning that it is likely that many more new magnetoelectric nanostructures will emerge to address any potential barriers which could emerge in the future. The most likely form would be a biodegradable nanostructure which could be self-eliminated on demand after accomplishing the main mission. For example, carbon nanostructures such as carbon nanotubes (CNTs) and graphene low-dimensional systems with magnetoelectric properties could become the building materials of the future medicine^59^.

## Materials and Methods

### Human ovarian cell culture and animal xenografts

Human ovarian carcinoma cell line (SKOV-3) (purchased from American Type Culture Collection (Manassas, VA)) was cultured in McCoy’s 5A medium (Life Technologies, NY) supplemented with 10% fetal bovine serum (Sigma-Alrich) and 1% penicillin-streptomycin (science-cell) in T25 flasks. All of the cells were maintained in a humidified-incubator at 37 °C temperature with 5% CO_2_. The confluent flasks were washed once with PBS buffer, and harvested by trypsinazation for 2 minutes. The harvested cells were neutralized using trypsin neutralizing solution, and the centrifuged at 1500 rpm for 5 minutes to precipitate the floating cells. The precipitated cells were re-suspended in McCoy’s 5A medium at a density of 1 × 10^7^ per ml. A 100 μl of the cell-suspension was injected subcutaneously on the right-thigh per each mouse (8 weeks old).

The current studies were performed on mice with inhibited immune system (nude mice). The mice, purchased from Taconic, were maintained under a specific pathogen-free environment in the institutional animal care facility. The tumor size was measured by a digital slide caliper and the tumor volume was calculated according to the following formula: volume = width^2^ × length/2. Tumors were measured every other day, and assigned a treatment type once the size reached approximately 200 mm^3^.

All the methods used in this study were carried out in accordance with the approved guidelines of the institutional animal care and use committee (IACUC) document # 13-045 at Florida International University (FIU).

### Fabrication of MENs

Details on the fabrication of toxicity-free MENs were described in our previously publications^36^,^40^. The basic structure of CoFe_2_O_4_–BaTiO_3_ coreshell 30-nm MENs was synthesized according to the following steps: 1) 0.058 g of Co(NO_3_)_2_·6H_2_O and 0.16 g of Fe(NO_3_)_3_·9H_2_O were dissolved in 15 mL of deionized (DI) water; 2) 5 mL of aqueous solution containing 0.9 g of sodium borohydride and 0.2 g of polyvinylpyrrolidone was added at 120 °C for 12 h to obtain CoFe_2_O_4_ nanoparticles; 3) BaTiO_3_ precursor solution was prepared by adding 30 mL of DI water containing 0.029 g of BaCO_3_ and 0.1 g of citric acid to 30 mL ethanolic solution containing 1 g of citric acid and 0.048 mL of titanium (IV) isopropoxide; 4) As-prepared CoFe_2_O_4_ nanoparticles (0.1 g) were added to the 60 mL of BaTiO_3_ precursor solution and sonicated for 120 min; 5) The resulted dispersed nanoparticles were dried on hot plate at 60 °C for 12 h, while stirring at 200 rpm; 6) The obtained powder was heated at 780 °C °C for 5 h in a box-furnace and cooled at 52 °C min^−1^ to obtain 30 nm-sized CoFe_2_O_4_–BaTiO_3_ coreshell MENs. To eliminate toxicity at least in *in vitro* studies, the nanoparticles were surface functionalized by a 2-nm thick coating of glycerol mono-oleate (GMO) according to the following steps: (i) GMO-MENs were prepared by incubating 0.1 mg of GMO with 5 mg of MENs in 5 mL of PBS (pH 7.4) buffer for 12 h; to achieve uniform surface modification, the solution was slowly agitated during incubation; (ii) The solution was centrifuged at 20000 rpm for 20 min at 10 °C to remove excess GMO; (iii) The obtained pellet was resuspended in ethyl acetate:acetone (70:30) solution and recentrifuged three times to obtain GMO-MENs. (iv) Surface-modified MENs were lyophilized and stored at 4 °C until further use. The particle size distribution was measured by a Zetasizer Nano series that uses the standard dynamic light scattering (DLS) approach. The measured zeta potentials for non-functionalized and functionalized (with GMO) MENs were −45 +/−1.72 mV and −41.6 +/−0.26 mV, respectively.

### Preparation of drug-loaded MENs

Previously, we described how surface functionalization of the nanoparticles was used to control the critical value of the a.c. magnetic field required to release the drug (PTX) off MENs in a wide range, from below 10 to over 200 Oe^40^. It was shown that coating MENs with a 2-nm GMO layer provided a release field of 50–100 Oe. This critical field is determined by a non-covalent interaction between oppositely charged GMO-coated MENs and PTX molecules in the bond (polarized due to the chemical interaction). It could be mentioned that the previous study also accounted for the binding efficiency after the drug-loaded MENs were introduced into the circulatory system. The nanoformulations were circulated in a pump-activated blood-flow-simulation environment for 2 hours at a 8-ml/minute rate, which reflected the human coronary flow rate per minute per gram of the myocardium. The nanoparticle surface functionalization was optimized to ensure drug loss of less than 10%. To obtain uniform binding, after 50 mg of PTX was added to a solution of 900 ml of the modified PBS (MPBS) buffer and 100 ml of GMO-coated MENs at a 5 mg/ml concentration, the solution was incubated for 3 hours while being stirred slowly. Then, the solution was centrifuged at 14,000 rpm for 10 minutes at 10 °C to remove any unbounded drug. The supernatant was isolated and absorbance was measured spectrophometrically at 230 nm using Cary-100 UV-VIS spectrophotometer.

To coat MENs with GMO, 1 mg of GMO was added to 5 mg of MENs in 5 ml of the PBS buffer. The mixture was then incubated for 12 hours while being slowly rotated in order to achieve uniform coating. Upon completion of the incubation process, the nanoparticles were centrifuged at 20,000 rpm for 20 minutes at 10° C. The pellet was washed in ethyl acetate:acetone (70:30) solution and re-centrifuged. The washing process was repeated thrice to completely remove the excess unbound GMO. Finally, the obtained pellet was lyophilized for 48 hours and stored for further use. The results of the energy dispersion spectrometry that depict the materials composition of GMO-MENs are summarized in Fig. S8.

### Preparation of PTX-loaded PLGA nanoparticles

PTX-loaded Polyoly(D,L-lactide-co-glycolide) (PTX-PLGA) nanoparticles were synthesized according to the emulsion solvent evaporation technique described elsewhere^60^. According to this procedure, 10 ml of organic phase (100 mg of PLGA, MW 5000, 5 mg of PTX, and 50 μl of triethyl-amine in 10 ml of dicloromethane) was added to 20 ml of aqueous phase (3% PVA). The saturated organic and aqueous phases were emulsified for 10 minutes using an ultrasonicator. The PLGA nanoparticle product was obtained after centrifuging the evaporated solvent at 20,000 rpm. The resulting nanoparticles were re-suspended in 2 ml of PBS (pH 7.4). HER2 conjugated PTX-PLGA nanoparticles were prepared by adding 1 mg of HER2, and 100ul of PTX loaded PLGA (10 mg/ml) to 400 ul of PBS (pH 7.4). To this solution, 2 mM of N-Hydroxysuccinimide, and 2 mM of 1-ethyl-3-(3-dimethylaminopropyl)carbodiimide were added. After 4 hours of incubation, HER2-conjugated PLGA nanoparticles were separated and quantified according to the procedure described previously^39^.

### Tissue preparation for H&E staining

Mice were euthanized by means of CO_2_ inhalation, immediately after which the tissues of interest were excised and stored in a 10% formalin solution overnight at room temperature. Tissues were processed using the VIP E 300 Tissue Tek Tissue processor SN 48940652 to create fixed, dehydrated, and cleaned cassettes. Tissues were transferred to warm paraffin filled molds using warm forceps and allowed to cool until solid. A Leica 2125 microtome was used to cut 4-μm sections, which were flattened by floating in a 40 °C water bath before being placed on VWR^®^ Superfrost^®^ Plus microscope slides to dry. Coverslips were added using Tissue Tek SCA Automated Coverslipper at this point, or after H&E staining using standard procedure, e.g. using the MMI H&E Staining Kit Plus. The sectioned tissue slides were imaged using Leica light microscope at 40X magnification.

### Tissue preparation for infrared imaging and EDS analysis

Mice were euthanized by means of CO2 inhalation, immediately after which the tissues of interest were excised and stored in a 10% formalin solution overnight at 4 °C. The tissues were cleaned under a stereomicroscope by removing hairs, excess fat, etc. A small piece of the tissue was cut and transferred into PBS, where it was rocked for 30 minutes with the PBS being replaced 3 times in 10 minute intervals to remove excess fixative. Tissues were carefully dried with a Kimwipe and transferred to a plastic mold containing OCT. Tissues were frozen either by immersing the mold directly into liquid nitrogen or in a bath of 2-methylbutane/liquid nitrogen at approximately −100 °C, depending on the type of tissue being processed. Frozen tissues were transferred to a −80 °C freezer for at least one night before being cut into 10 μm sections with a Leica CM3050 Cryostat. Finally, tissue slices were mounted on VWR^®^ Superfrost^®^ Plus microscope slides and dried on a slide warmer at 37 °C for 1–2 hours. Slides were then ready to use for EDS analysis, Infrared imaging with immunostaining, and/or H&E staining. For immunostaining, the tissue sectioned slides were stained using HER2Sense^TM^ 645 fluorescent imaging agent to visualize HER-2 (Human Epidermal Growth Factor Receptor 2) overexpression. Immunostained samples were prepared by incubating the cross-sectioned tissue slides with HER2Sense (μM) for 15 minutes at room temperature. After incubating the samples for 15 minutes, tissues were washed three times using PBS (pH = 7.4) and mounted with ProLong-Gold anti-fade reagent. Flourescent imaging was performed using Leica fluorescent microscope with Cy5 filter at 40X magnification.

### Magnetic-field dependent Zeta Potential

The magnetic field dependence of the MENs’ surface charge was measured using Malvern Zetasizer. To simulate the charge of MENs in the blood, 0.5 mg of MENs were dispersed in a 1-ml cuvette containing PBS buffer with a pH level of 7.3, which is similar to the pH level of blood. The cuvette was exposed to a uniform magnetic field ranging from 0 to +/−100 Oe with a 10-Oe increment using an electromagnet. The effect didn’t depend on the polarity of the field.

### Cell lysate preparation for further AFM/MFM imaging

Cells were seeded with a density of 0.7 to 1.0 × 10^6^ cells per T-25 flask and incubated overnight. Cell cultures were maintained by changing the media 2–3 times per week, until flasks reached confluence. Before each experiment, the media was removed and replaced with 3 mL of the desired drug form, and incubated with their respective field treatment (DC field, no field, and/or DC+AC field, respectively). Upon completion of the field treatment, the cells were removed from the incubator and the media was discarded. The amount of free drug in the cell lysate was measured to determine the amount of the drug absorbed and released inside the cell. After media was discarded, cells were trypsinized and washed thrice with ice cold PBS, then resuspended in 1 mL of DMSO, and incubated for 1 hour at 37 °C. After 1-hour incubation, the solution was vortexed and centrifuged at 4,500 RPM for 10 minutes, to pellet the cellular debris and obtain the lysate in the supernatant. The cell lysate was collected and measured spectrophometrically in scan mode. A standard curve was generated by resuspending known concentrations of drug in DMSO and diluting it in 1 mL of PBS. Each peak absorbance at a specific wavelength on the curve corresponds to a known concentration of drug. The cell lysate shows peaks at similar wavelengths, indicating that there is free drug in the cell lysate. The amount of drug in cell lysate is dependent of the number of cells in the sample. The amount of cells in the sample was normalized by measuring the protein content of that sample. The protein content of the cell lysate was determined immediately after collection to avoid protein degradation using Bio-Rad Protein Assay Kit (Bradford method) and measuring the absorbance in the range of 587–591 nm. All spectrophotometric measurements were performed by Cary 100 UV-VIS spectrophotometer.

### AFM and MFM imaging

Scanning probe microscope (SPM) Multimode was used to measure AFM and MFM images of cell lysates. The images were obtained with a CoCr-based “hard” magnetic nanoprobe in a lift mode at a scan height of 10 nm. According to this mode, each image line is scanned twice, first measuring the topography through the Van der Waals interaction (data type: height, Z range: 75 nm) and then measuring the magnetic signal through the magnetic force by lifting the probe at the scan height distance (data type: phase, Z range: 50^0^). The magnetic coercivity and the saturation magnetization of the MFM probe are believed to be on the order of 500 Oe and 500 emu/cc, respectively.

### STS measurements of I-V curves

STS function of scanning tunneling microscopy (STM) mode of Multimode was used to obtain I-V curves of a direct point contact between an STM nanoprobe and a MEN in presence of a d.c. magnetic field in a 200-Oe range along the central orientation. The magnetic field was generated by a customized multi-turn coil wrapped around the sample.

### Vibrating sample magnetometry

A room-temperature Lakeshore vibrating sample magnetometer (VSM) with a 3-T magnetic field sweep was used to measure key magnetic properties of nanoparticles under study including the magnetization saturation and the magnetic coercivity.

### Transmission electron microscopy (TEM)

Phillips CM-200 200 kV Transmission Electron Microscope (TEM) with Energy Dispersive Spectroscopy (EDS) option was used to obtain TEM images and EDS profiles.

### Scanning electron microscopy (SEM) and Energy-dispersive Spectroscopy

JEOL- JIB 4500 multibeam system (FIB/SEM) with a Thermo-scientific Noran system 7 was used to obtain SEM images and carry out the EDS analysis.

## Additional Information

**How to cite this article**: Rodzinski, A. *et al.* Targeted and controlled anticancer drug delivery and release with magnetoelectric nanoparticles. *Sci. Rep.* **6**, 20867; doi: 10.1038/srep20867 (2016).

## Supplementary Material

Supplementary Information
