# Supplementary material for: Targeted and controlled anticancer drug delivery and release with magnetoelectric nanoparticles
Source: Sci Rep. 2016 Feb 15;6:20867. doi: 10.1038/srep20867 (PMC4753509; doi:10.1038/srep20867)
Supplement: Supplementary Information [file srep20867-s1.pdf]

Supplementary Information

Title: Targeted and controlled anticancer drug delivery and release with magnetoelectric nanoparticles

Authors: Alexandra Rodzinski[1], Rakesh Guduru[1], Ping Liang[2,3], Ali Hadjikhani[4], Tiffanie Stewart[1], Emmanuel Stimphil[4], Carolyn Runowicz[5], Richard Cote[6,7], Norman Altman[6], Ram Datar[6,7], and Sakhrat Khizroev[1,4*]

Affiliations

[1]Department of Cellular Biology and Pharmacology, Herbert Wertheim College of Medicine, Florida International University, Miami, Florida 33199

[2]Electrical and Computer Engineering, University of California, Riverside, CA 92521

[3]Cellular Nanomed Inc., Weston, FL 33331

[4]Department of Electrical and Computer Engineering, Florida International University, Miami, FL 33174

[5]Department of Obstetrics and Gynecology, Herbert Wertheim College of Medicine, Florida International University, Miami, Florida 33199

[6]Department of Pathology, Miller School of Medicine, University of Miami, Miami, FL 33136

[7]John T. Macdonald Foundation Biomedical Nanotechnology Institute, University of Miami, Miami, FL 33136

*To whom the correspondence should be addressed:

Sakhrat Khizroev, Department of Cellular Biology and Pharmacology, Herbert Wertheim College of Medicine, Florida International University, Miami, FL, Ph. 305-348-3724, E-mail: khizroev@fiu.edu

Supplementary Information Figure Legends

Figure S1: Direct measurements of the cancer cell lysate content.
Figure S2: AFM study of polymer nanoparticles.
Figure S3: A cured mouse throughout the treatment.
Figure S4: AC-Field setup.
Figure S5: Biodistribution of MENs.
Figure S6: Imaging of H&E stained tissues sections.
Figure S7: Imaging of tissue sections with Her2Sense 645 fluorescent agent.
Figure S8: Energy dispersive spectroscopy of MENs.
Figure S9: Control nanoparticles and control electroporation setting.

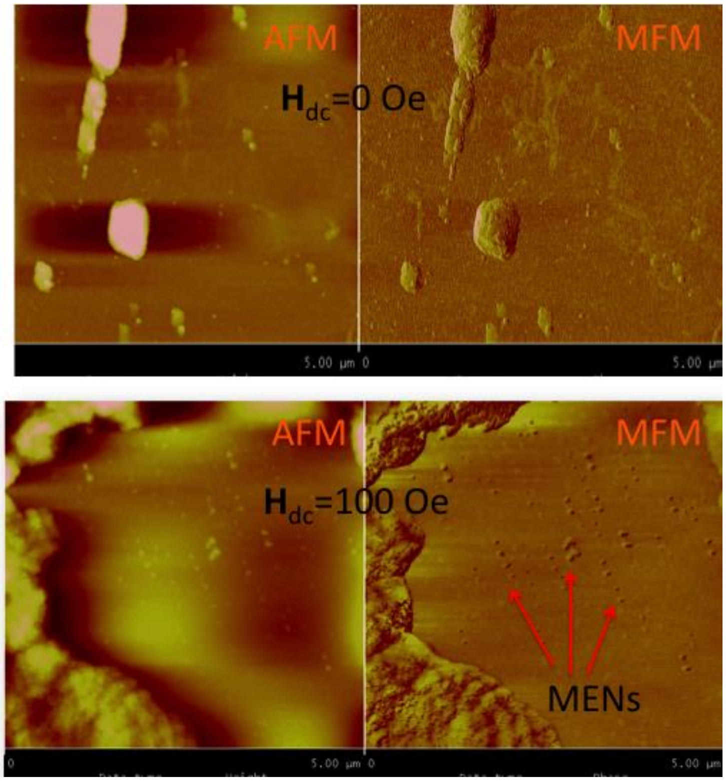

A

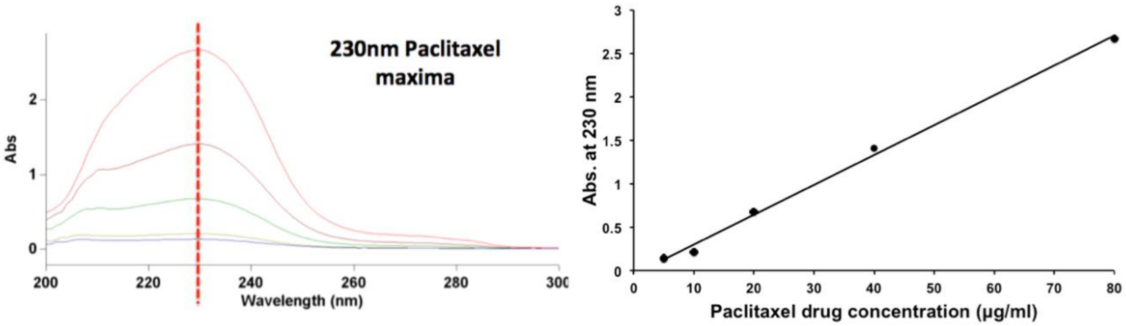

B

Figure S1: Direct measurements of the cancer cell lysate content. (A) Atomic force microscopy (AFM) and magnetic force microscopy (MFM) images of cancer cell lysate prepared from the media with MENs (top) before and (bottom) after application of a 100-Oe d.c. magnetic field. (B) The spectrophotometrically measured absorption maxima and standard linear calibration curve for PTX at different drug concentrations.

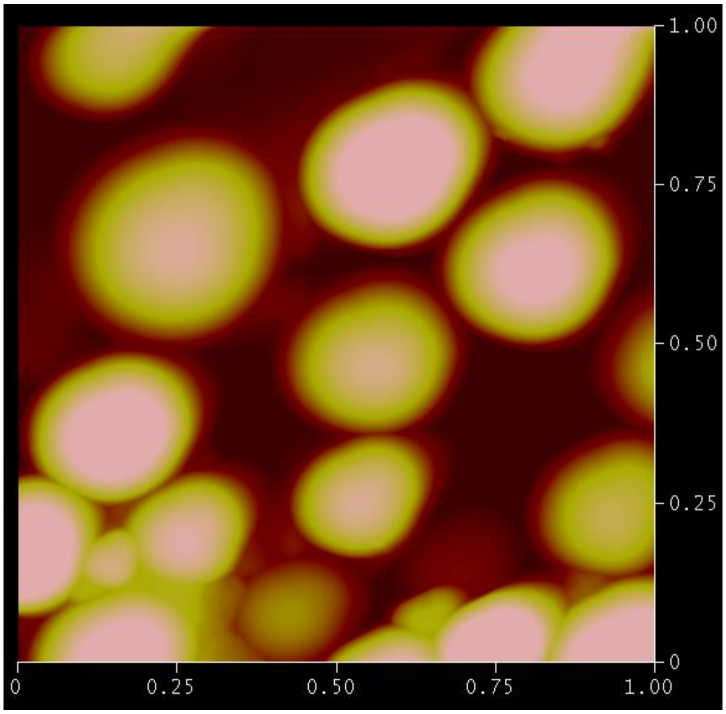

Figure S2: AFM study of polymer nanoparticles. AFM image of 200-nm PLGA nanoparticles.

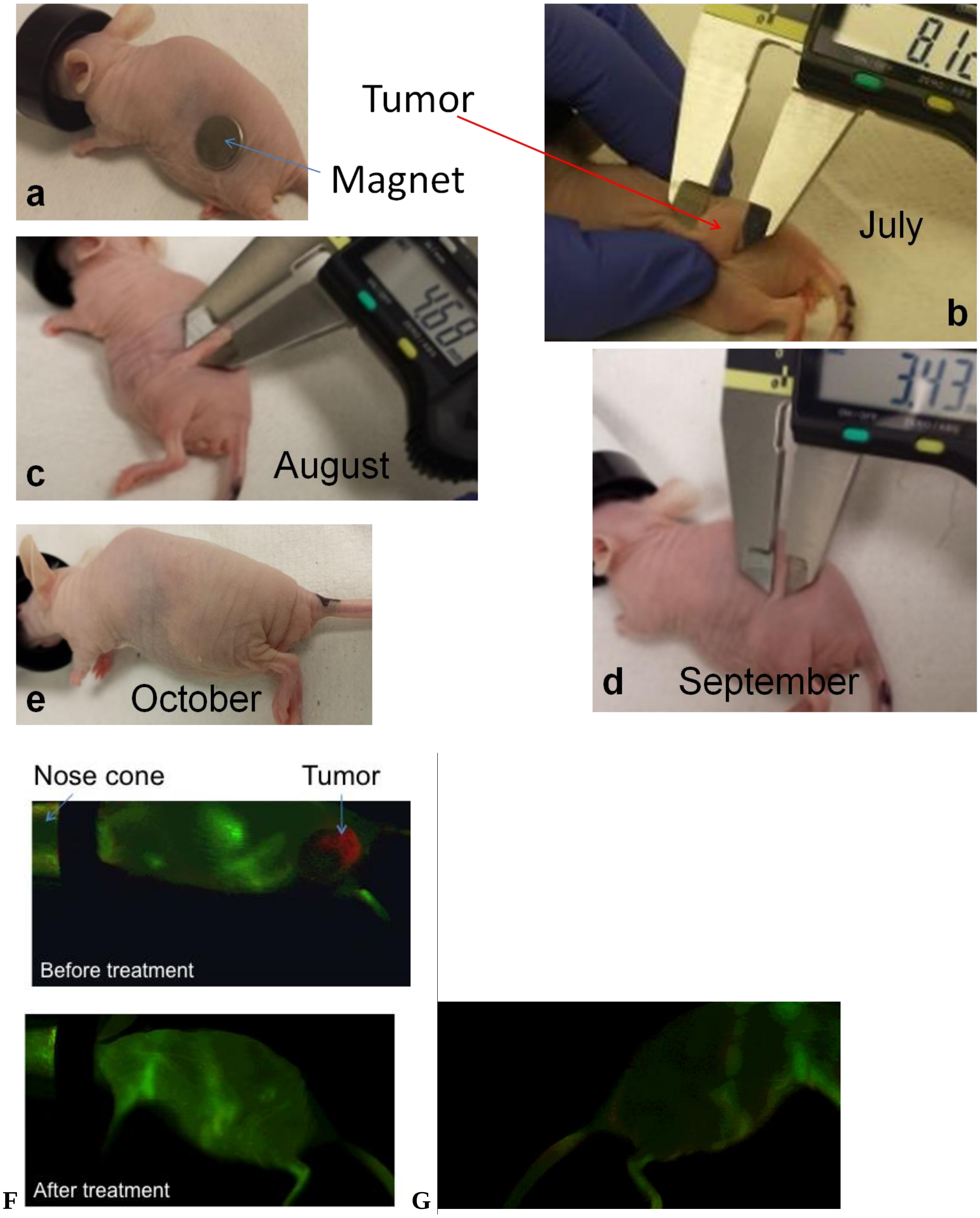

Figure S3: A cured mouse throughout the treatment. (A) A magnet is attached for one hour immediately after a weekly injection. The tumor size on (B) July 11: 268 mm^3, (C). August 14: 51 mm^3, (D) September 5: 20 mm^3, (E) October 13: no visible tumor. (F) IR images (with fluorescent agent Her2Sense 645 taken before (top) and after the completion of the MEN treatment. The agent had excitation and emission maxima at 643 and 661 nm, respectively. (G) IR image for a mouse with a tumor after an injection with conventional magnetic nanoparticles, FNs, conjugated with the same fluorescent agent.

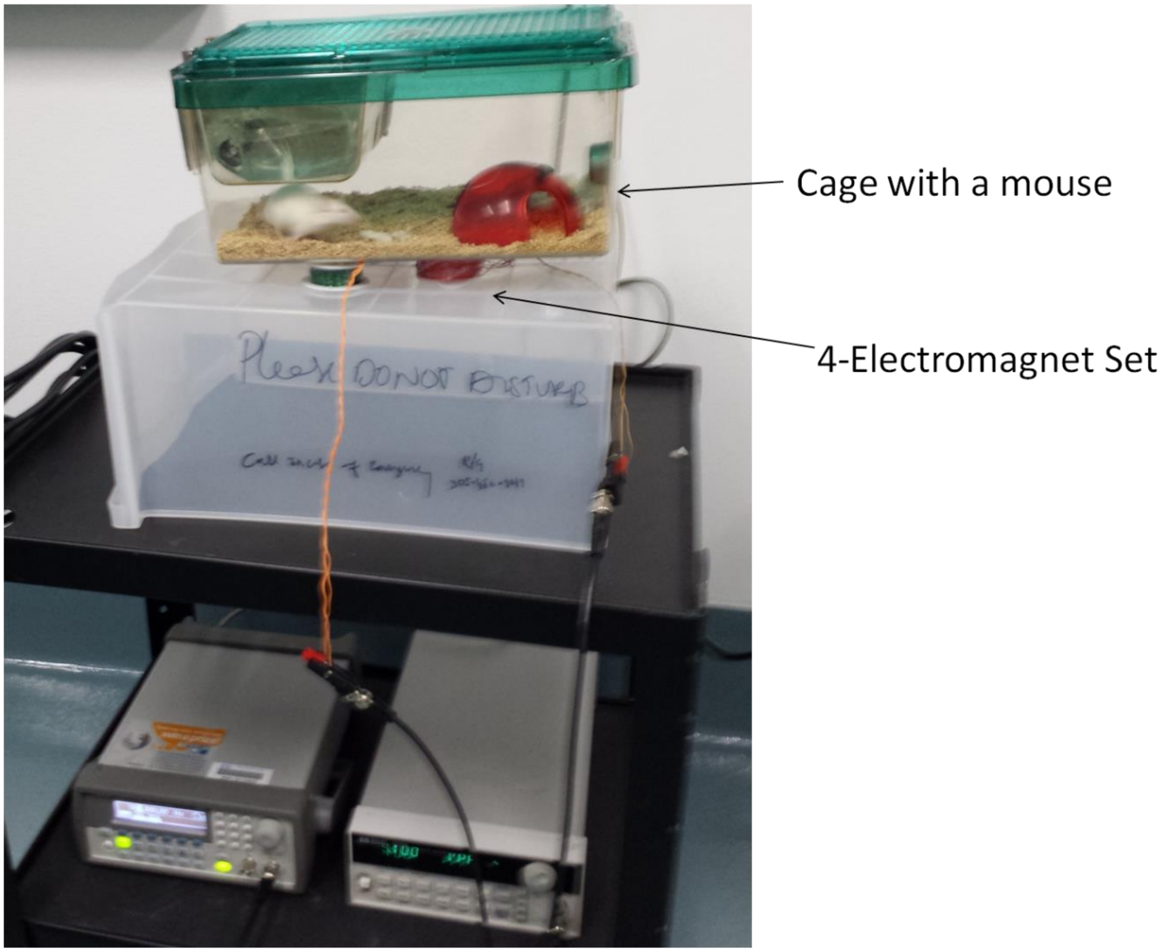

Figure S4: AC-field setup. There are four coils placed directly under the cage to generate an a.c. field in the cage.

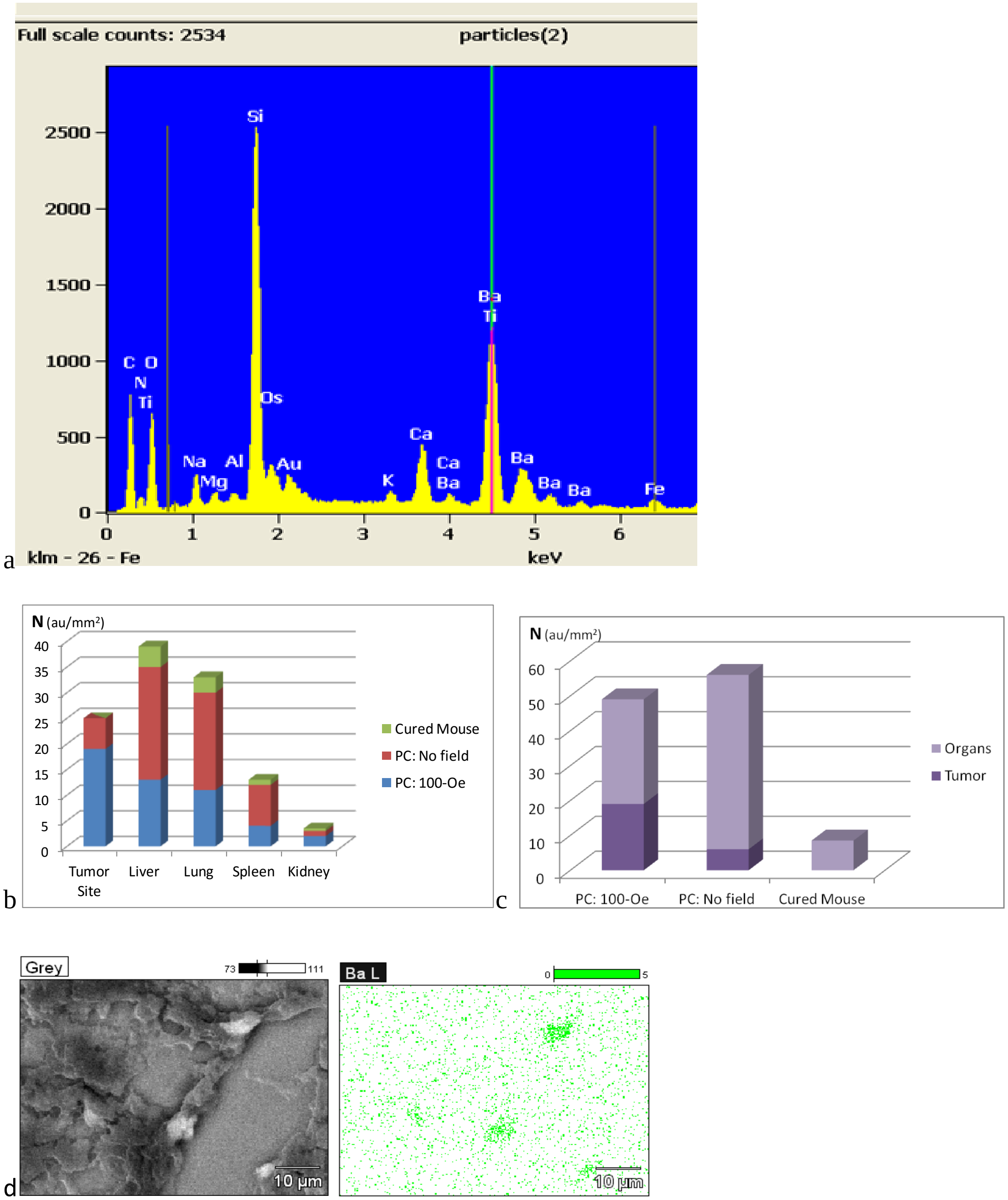

Figure S5: Biodistribution of MENs. (**a**) Chemical compositional spectrum from tumor sections in euthanized mice after initial treatment (measured through EDS). (**b)** The areal concentration of MENs in the tumor site and different organs including liver, lung, spleen, and kidney for three mice under study including (i) a cured mouse and two positive control mice which were euthanized only two weeks after starting MEN-PTX weekly treatments (ii) in a 100-Oe field (PC: 100-Oe), and (iii) without application of any field (PC: No field). (**c**) The concentration of MENs in the tumor and all the four organs combined for the three euthanized mice under study. (**d**) A SEM image of a spleen region (left) and an EDS-Ba image of the same region which confirms the presence of MENs in a positive control mouse which was treated by MENs in the field environment.

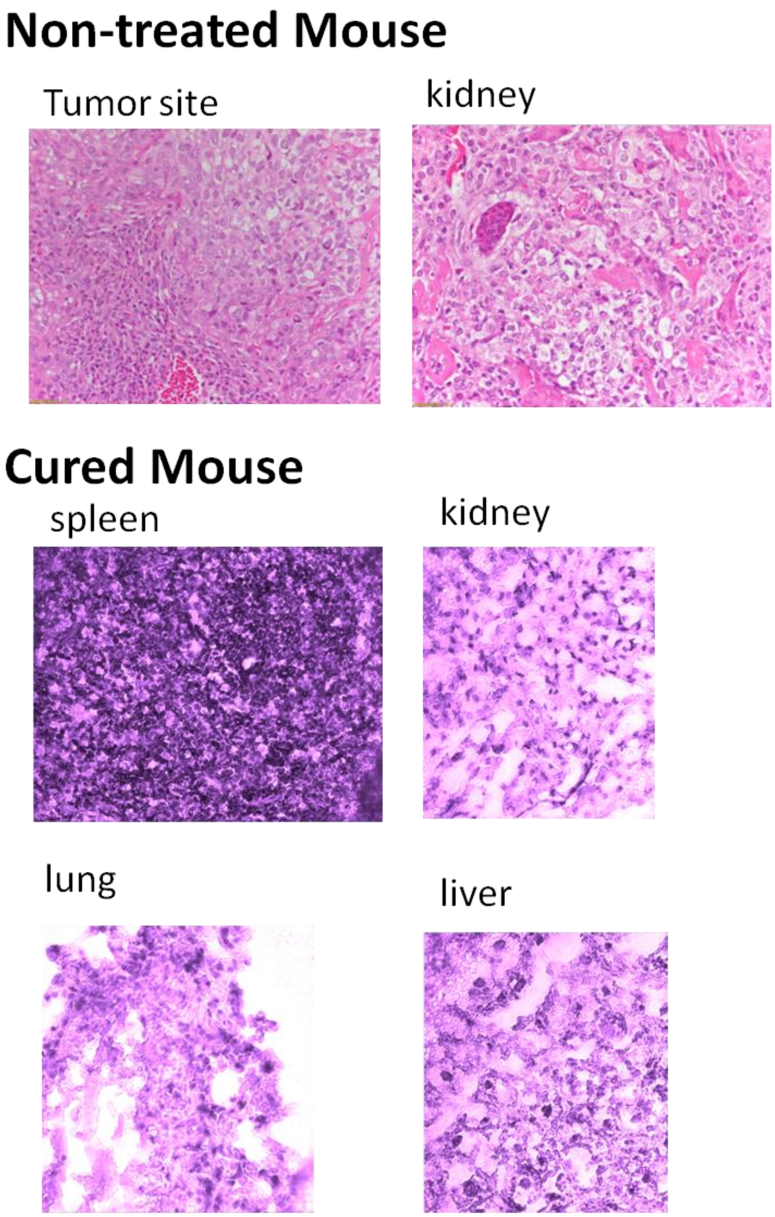

Figure S6: Imaging of H&E stained tissues sections. (top) Images which show cancer cells in the tumor site and metastasized cancer cells in the kidney of an untreated mouse. (bottom) Images which show normal cells in different organs, including liver, lung, spleen, and kidney, or a cured mouse.

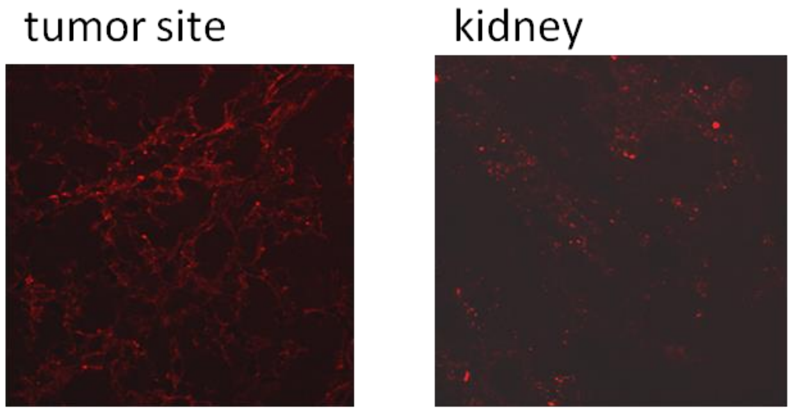

Figure S7: Imaging of tissue sections with Her2Sense 645 fluorescent agent. Images of cancer cells in the tumor site and metastasized cancer cells in the kidney of an untreated mouse. Note: No contrast can be detected in tissue sections from different organs of a cured mouse.

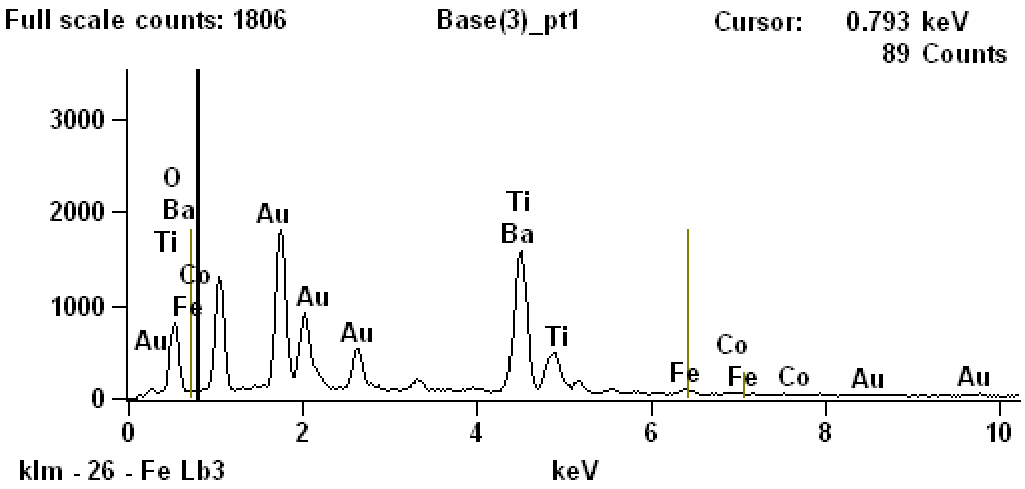

a.

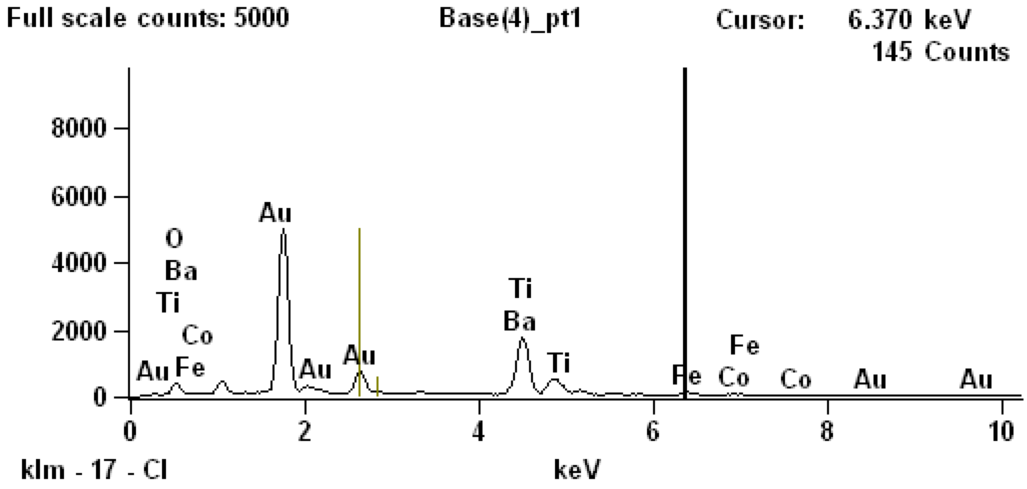

b.

Figure S8: **Energy dispersive spectroscopy of MENs**. Energy dispersion spectroscopy (EDS) results depict the composition of **(a)** naked MENs and **(b)** GMO-coated MENs.

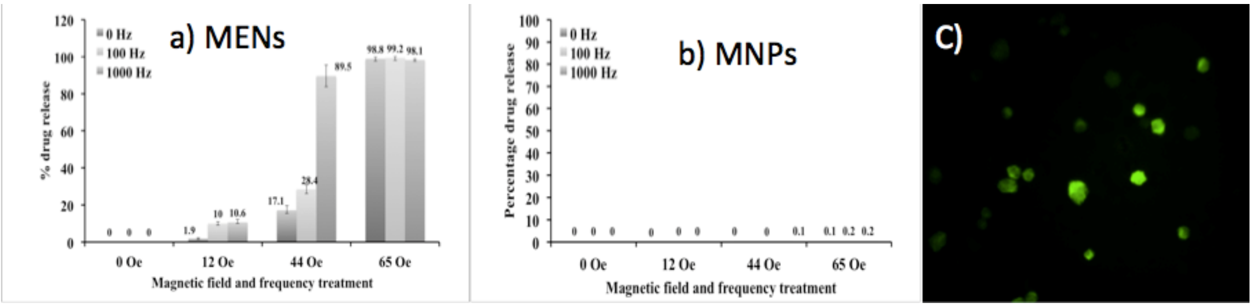

Figure S9: Control nanoparticles and control electroporation setting. The drug release percentage for a range of fields and frequencies including 0, 12, 44, and 65 Oe, and 0, 10, and 100 Hz. MNPs for (a) MENs and (b) conventional 10-nm iron oxide magnetic nanoparticles (MNPs). MNPs display no ME effect. (c) Confocal microscopy imaging of the released drug (off MENs) via application of a remote pure electric field (of 1000 V/cm). No magnetic field has been applied.
